# Effects of Mutations in TSC Genes on Neurodevelopment and Synaptic Transmission

**DOI:** 10.3390/ijms22147273

**Published:** 2021-07-06

**Authors:** Davide Bassetti, Heiko J. Luhmann, Sergei Kirischuk

**Affiliations:** Institute of Physiology, University Medical Center of the Johannes Gutenberg University, Duesbergweg 6, 55128 Mainz, Germany; luhmann@uni-mainz.de (H.J.L.); kirischu@uni-mainz.de (S.K.)

**Keywords:** tuberous sclerosis, development, synaptic transmission, GABA, glutamate, excitation-to-inhibition balance, neuronal network hyperactivity

## Abstract

Mutations in TSC1 or TSC2 genes are linked to alterations in neuronal function which ultimately lead to the development of a complex neurological phenotype. Here we review current research on the effects that reduction in TSC1 or TSC2 can produce on the developing neural network. A crucial feature of the disease pathophysiology appears to be an early deviation from typical neurodevelopment, in the form of structural abnormalities. Epileptic seizures are one of the primary early manifestation of the disease in the CNS, followed by intellectual deficits and autism spectrum disorders (ASD). Research using mouse models suggests that morphological brain alterations might arise from the interaction of different cellular types, and hyperexcitability in the early postnatal period might be transient. Moreover, the increased excitation-to-inhibition ratio might represent a transient compensatory adjustment to stabilize the developing network rather than a primary factor for the development of ASD symptoms. The inhomogeneous results suggest region-specificity as well as an evolving picture of functional alterations along development. Furthermore, ASD symptoms and epilepsy might originate from different but potentially overlapping mechanisms, which can explain recent observations obtained in patients. Potential treatment is determined not only by the type of medicament, but also by the time point of treatment.

## 1. Introduction

**Figure 1:**
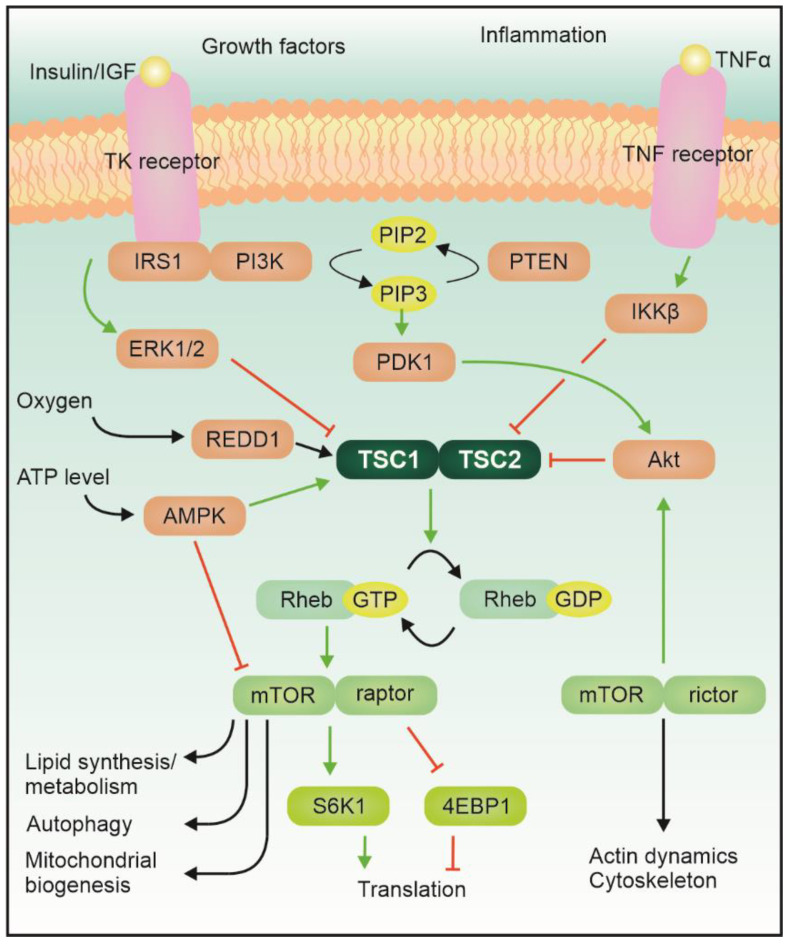
mTOR signaling pathway. Graphical depiction of the main upstream regulators of Tsc1–Tsc2, as well as how it can influence the mTOR pathway. For details see text.

Tuberous sclerosis complex (TSC) is an autosomal dominant genetic disease featuring localized uncontrolled growth of tissues, known as hamartomas, in many organs, including the central nervous system (CNS) [1]. A large proportion of the cases are sporadic mutations (ca. two thirds [2]). The disease results from a loss-of-function mutation in either the TSC1 or TSC2 gene, which encode for hamartin and tuberin proteins, respectively; two proteins that together form the Tsc1–Tsc2 complex. This complex, which is the converging center of several signaling pathways, has GTPase activity and can inhibit the activity of Ras homolog enriched in the brain (Rheb). The complex itself is modulated by phosphorylation via, for example, Akt or AMPK (Figure 1, [3]).

TSC1 or TSC2 inactivation causes an increase of GTP-bound Rheb, which is a regulator of the mechanistic Target of Rapamycin pathway (mTOR) activity, effectively leading to one of the key characteristics of the TSC disease: an upregulation of the mTOR pathway activity. The mTOR protein itself is a serine/threonine kinase which acts as an integrator of various signals belonging to different modalities, related for example to the energetic status of the cell and the availability of growth factors and nutrients, and regulates the growth program of the cell [4]. mTOR, together with other components, can interact with the regulatory associated protein raptor to form a rapamycin-sensitive complex (mTORC1) or with the rapamycin insensitive companion of mTOR (rictor), to constitute the mTOR complex 2 (MTORC2). Activation of mTORC1 leads to phosphorylation of its main downstream targets (including 4e binding protein 1 and S6 kinase), which control local protein synthesis, cellular growth, and autophagy [5]. The level of mTORC1 hyperactivation in TSC correlates well with the severity of the symptoms. mTORC2, instead, mostly regulates actin cytoskeleton dynamics via PKC or small GTPases of the Rho family. Furthermore, it can activate Akt, which is an inhibitor of the activity of the Tsc1–Tsc2 complex [6]. Albeit the role of mTORC2 in other diseases featuring mTOR hyperactivation is becoming more prominent, in TSC its contribution remains elusive [7].

In the central nervous system, the mTOR pathway is crucial for a variety of functions, including cell growth, local protein synthesis, synaptogenesis and synaptic pruning. Alteration of mTOR activity results in neurological effects, like epilepsy, intellectual disability, and autism spectrum disorder (ASD) (for review, see [8,9,10]). Additionally, TSC patients often report a number of other conditions including overactivity, impulsivity, anxiety, mood swings, aggressivity, and less often self-injury, obsessions and psychosis. This set of further symptoms takes the collective name of Tuberous-sclerosis Associated Neuropsychiatric Disorders (TANDs, see [11]). It is therefore not surprising that this disease can have a strong impact on patients’ life [12].

## 2. Neurodevelopment, Epilepsy, and Autism

The complex nature and the large number of different factors at play makes tuberous sclerosis particularly impervious to the establishment of a clear set of causes and effects so far. In TSC patients, hyperactivation of mTOR and the consequent inflammation start at the fetal stage, at which it is already possible to observe morphological alterations at the cellular and tissue level [13]. Therefore, neurological manifestations in the CNS temporally precede the appearance of symptoms, and this is particularly noticeable in humans with large-scale lesions. Different types of lesions occur in the brain of affected patients. The most common form are cortical tubers, which are areas of dysplastic tissue lacking correct lamination and containing enlarged cells. Other manifestations are subependymal nodules (SEN) and giant cell astrocytomas (SEGA), which are areas enriched in astrocytic cells. They appear in the vicinity of ventricles and can in some cases lead to hydrocephalus by occlusion of cerebral liquor circulation (for review [14,15]). The alterations are not restricted to those, as closer inspection reveals localized alterations, with smaller scale, also outside tuberal tissue [16].

The main neurological manifestations of TSC include intellectual disability, epilepsy, and autism. Autism spectrum disorder (ASD) is a neurodevelopmental set of conditions that features reduced sociality, repetitive/stereotyped behaviors and cognitive inflexibility. The prevalence of ASD is around 1.5–1.7% (although with large variation depending on factors such as country and diagnostic criteria, see [17]). The pattern of ASD symptoms diagnosed in TSC individuals resembles the one observed in non-syndromic cases [18]. This condition typically develops at an early age and is diagnosed around the third year [19]. However, it is frequently recognized by parents about one year earlier [20]. Concerning children affected by TSC, neuropsychological tests performed at 12 months can identify the risk of developing ASD [21] and even earlier predictions can be performed, based on EEG activity already at 12 weeks [22]. Over the first two years of life, the brain of TSC individuals with ASD shows alterations in white matter [23]. It is therefore clear that changes in neurophysiology and in behavior begin long before the ASD diagnosis.

Epilepsy is an even more common manifestation of TSC, and several types of seizures are present in the majority of the cases (around 86% of patients [24]). Generally, mutations in TSC2 tend to lead to a more severe epileptic phenotype compared to mutations in TSC1 [25]. Tubers or perituberal tissue are considered the preferential epileptogenic zones [26]. However, a recent stereo-EEG study demonstrated that one half of the cases display a focal epileptogenic zone located in a dominant tuber, but the other half show a complex organization involving tissue with normal appearance (and also not perituberal) [27].

The interaction between tubers, seizures, cognitive functions and ASD in TSC during development is still elusive and requires further investigations. The association between epilepsy and ASD appears to be the most established; in fact early epilepsy constitutes a risk factor for ASD and intellectual deficits in individuals with TSC (for a review, see [28]). Although early life seizures have an effect on intellectual disability, TSC individuals tend to display decreased IQ compared to unaffected siblings also in the absence of seizures [29]. Age of seizures onset can have an effect on ASD symptoms, but seizures do not seem to be necessary for their appearance. In fact, 21% of the children at risk of ASD and 11% of the ones with developmental delay at 24 months have no previous epilepsy history [30], and fetal lesions revealed by MRI imaging correlate with cognitive developmental deficits, but not with epilepsy [31]. Nevertheless, alterations in EEG appear to precede the onset of epileptic events, thus further pointing to the high importance of the early postnatal neuronal network functioning for subsequent development [32].

**Figure 2:**
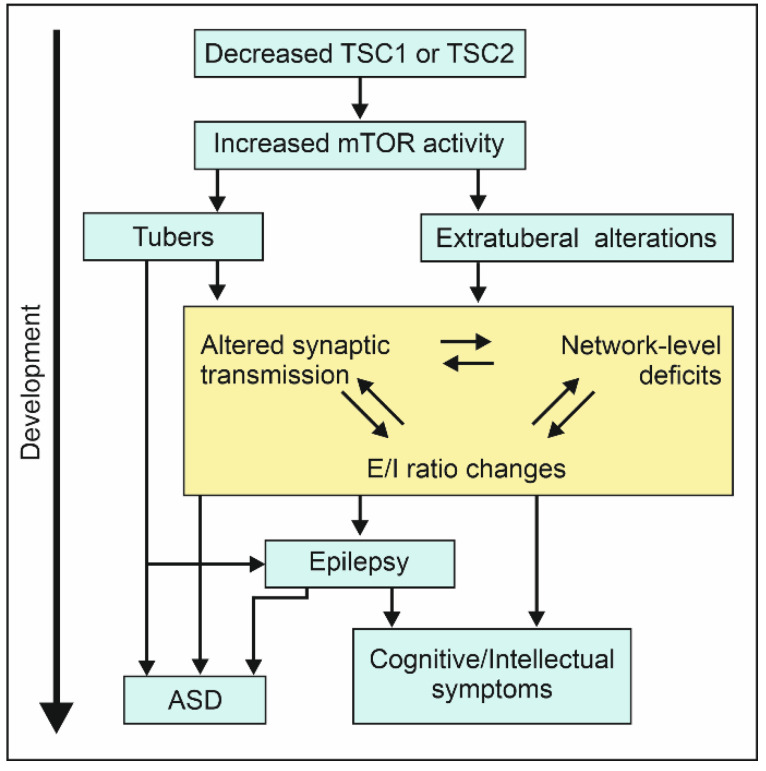
Suggested interplay between different factors during development. Graphical representation of how mutation of TSC1 or TSC2 and increased mTOR activity are associated to neurological symptoms. Altered mTOR activity translates into formation of tubers, as well as a set of consequences which include extratuberal alterations and lead to network level alterations, altered synaptic transmission and E/I ratio changes. This can lead to the development of epileptic seizures from early age. Cognitive and behavioral symptoms might arise from the seizures, or the set of symptoms.

Those considerations put emphasis on the interaction between different factors and their time course during development in TSC (Figure 2). Murine models are promising tools available for appropriate studies. In fact, data obtained from rodents allows understanding how mutations in Tsc genes can affect neurodevelopment in terms of both morphological alterations and network activity changes, and thus unraveling candidate pathological developmental changes to be alleviated in TSC patients.

## 3. Mouse Models and Development

Several mutant mouse lines of TSC have been generated with various outcomes in terms of brain lesions, seizures, and phenotype. Table 1 represents a list of models that cover mutations in different cell populations, and in the following sections we summarize some of the available results, mainly focusing on the developmental aspects of synaptic functions in the CNS.

Homozygous mutations in either Tsc1 or Tsc2 are not viable [33,34]. Heterozygous Tsc1 and Tsc2 mice do not display brain lesions, and Tsc2^+/−^ rats feature cortical tubers, albeit with low incidence [35,36]. No spontaneous seizures were detected in these models, beside in Tsc1^+/−^, which undergoes a transient period of epileptiform activity at young age [37]. Interestingly, those seizure resemble what is observed in human [38]. While all three models present with varying degrees of behavioral and cognitive deficits [39,40,41,42,43,44,45,46], their relationship with the rich variety of manifestations observed in ASD and TANDs symptoms in humans is not necessarily straightforward.

The lack of spontaneous epileptic seizures in some rodent models allows study of the relationship between alterations in brain morphology during development and the observed phenotype. Pharmacological induction of seizures during early developmental stages (P7 and P14) in WT rats causes the appearance of social interaction deficits at 3 to 6 months of age. However, the pattern of deficits of WT rats that underwent kainate-induced seizures and that of Tsc2^+/−^ rats are not completely overlapping. These results suggest that both haploinsufficiency of Tsc2 without epileptic activity and transient epileptic seizures during early postnatal development can lead to ASD symptoms in later life. It remains an intriguing question whether similar mechanisms underlie manifestation of ASD in these two cases [45].

With the use of modern genetic tools several mouse lines have been generated in which either Tsc1 or Tsc2 had been deleted in specific cell lines or with selective or sparse loss of function. Furthermore, it is possible to recreate in mice an overall heterozygosity and with double loss of function restricted to specific cell types. Some of those models present spontaneous seizures and several features which can be compared to those observed in patients, such as enlarged cells or alteration of lamination (see Table 1). Furthermore, there is the possibility to induce the recombination at specific time points, which allows investigation of the age-dependent effect. As an example, lack of Tsc1 in glial fibrillary acidic protein (GFAP) expressing cells can lead to spontaneous seizure, but only if the deletion was performed at early ages (2 weeks), and not later (6 weeks of age) [47].

## 4. Morphological Alterations

The Tsc1–Tsc2 complex regulates morphological properties of cells via mTOR signaling. mTORC1 phosphorylates the ribosomal S6 kinase and translational regulator 4E-BP1, thus stimulating protein synthesis and cell growth, and mTORC2 plays a role in the cytoskeleton organization. Moreover, mTOR activity appears to regulate not only the temporal aspects of cell growth but also its spatial features, i.e., where and how the cell grows [60]. Interestingly, monoallelic loss of either Tsc1 or Tsc2 does not result in cortical tuber-like formation in mouse brain both in Tsc1^+/−^ [43] and in Tsc2^+/−^ mice [61]. Loss of both copies of the gene appears to favor the formation of larger morphological alterations. For example, human induced pluripotent stem cells were obtained by reprogramming fibroblast from a heterozygous patient and a conditional second allele deletion was added. The resulting 3D cortical spheroid displayed strong alteration only in cells with “second-hit” and not in unaffected cells in their vicinity [62]. Significant efforts have been devoted to generate models that could recapitulate or mimic brain lesions observed in patients, and to analyze the contribution of specific cell types to morphological alterations.

Deletion in-vivo of Tsc1 in almost all neurons at the age of embryonic day 12.5 (E12.5), together with hemizygosity in the remaining cells, led to morphological cell changes that resemble human TSC phenotype. Many cortical neurons were dysplastic and had increased cellular size. These mice demonstrated developmental deficits from postnatal day 5 (P5), spontaneous seizures, and shortened lifespan (about 35 days). No alterations have been observed in astrocytes. In addition, this mouse line showed strong hypomyelination [48]. A similar investigation was performed using in utero electroporation at E15–16, resulting in sparse double deletion of Tsc1. This intervention led to structural tuber-like formations and reduced threshold of seizures in adult animals [63].

In both of the above-mentioned studies, no sign of astrogliosis has been observed. However, glial cells could play a role in TSC, as selective deletion of Tsc1 in astrocytes causes epileptic seizures already after the first postnatal month. In parallel, it led to increased brain size, and the observed astrogliosis was accompanied by aberrantly located pyramidal neurons in the hippocampus, although cortical tubers or neocortical lamination deficits were not observed. Thus, specific Tsc1 disruption in astrocytes is sufficient to cause neuronal abnormalities and epilepsy [64]. Moreover, a recent study showed that the mere exposure to astrocyte-conditioned medium from TSC cultures was sufficient to affect dendritic arborization and the number of vesicular GABA transporter (VGAT) positive synapses in cultures, indicating that Tsc1-deficient astrocytes can influence functional and morphological maturation of neurons through release of biologically active substances [65].

Tsc1–Tsc2 complex does not only influence cell migration and growth, but it also plays a crucial role in axon formation. Overexpression of Tsc1 or Tsc2 in hippocampal cultures reduced axonal growth, while their knockdown caused the appearance of many cells with multiple axons [66]. Similarly, Tsc2 haploinsufficiency affects axon guidance in vivo as Tsc2^+/−^ mice displayed abnormal retinogeniculate projections [67]. Despite the observed hypomyelination, mTOR activity was not elevated in oligodendrocytes in Tsc1^c/-^;SynI CKO mice, indicating that neuronal deficits can per se lead to disturbed myelination [48]. On the other hand, conditional knockout of Tsc2 in oligodendrocyte precursor cells (OPCs) under the Olig2 promoter also resulted in a strong hypomyelination, together with reduced oligodendrocyte number and astrogliosis. It was suggested that elevated mTOR activity in OPCs may lead to a reduction in myelination via a switch in the fate of OPCs from oligodendrocytic to astrocytic lineage [57]. Deletion of one copy of Tsc2 in radial glial cells caused cortical enlargement, lamination defects, and enlarged size of both neurons and astrocytes. Although the number of oligodendrocytes was instead increased, this model also featured strong hypomyelination [54]. In parallel with the induction of epileptic activity, potentiated mTOR activity in astrocytes led to activation of microglia. However, the blockade of microglia activation could not prevent the occurrence of epileptic seizures, suggesting that it might be a secondary effect [68]. TSC patient-induced pluripotent stem cells (iPSC)-derived neuronal mono-cultures showed increased dendritic branching, as well as action potential firing. However, neuronal defects were more apparent in TSC neuron–oligodendrocyte co-cultures, confirming the importance of neuro–glia interaction in this model system [69]. Thus, development of TSC symptoms in vivo is regulated not only by neurons and/or neuronal activity but appears to involve functional neuro–glial interactions.

To exclude possible epilepsy-induced functional changes, analysis of specific effects on cell morphology and functions has been performed in hippocampal primary neuron or organotypic slice cultures from Tsc1C mice, in which a CRE-mediated conditional deletion of Tsc1 was performed in a restricted subpopulation of neurons. Both Tsc1 and Tsc2 deficient pyramidal cells demonstrated enlarged soma size. In addition, spine size and density were increased, suggesting that Tsc1 or Tsc2 deletion did not only change somatic parameters but could modify dendritic structure and synaptic inputs. Monoallelic loss of Tsc1 produced changes in spine properties comparable to the complete ablation of Tsc1 [70].

However, in vivo, the effect on spines shows a clear time dependence. In Tsc2^+/−^ mice spine density in layer 5 pyramidal neurons was comparable to that in WT mice at P19-20, but was significantly increased at P29–30, indicating a diminished synaptic pruning [40]. It should be kept in mind that while spine stabilization can depend on Rheb activity (and in turn on Tsc1–Tsc2), some Tsc-mediated effects may be mTOR-independent [71]. Similarly, altered expression of actin-crosslinking protein filamin A could increase dendritic complexity in Tsc1 deficient mice in an mTOR-independent and ERK1/2-dependent manner [72].

The wide range of morphological changes observed in different TSC models include alterations in axon guidance, dendritic arborization and spine number and stability. Thus, a correct interplay between different cell types are prerequisite for proper brain development and Tsc1 or Tsc2 mutations probably leads to functional changes in synaptic communications (but see [73]).

## 5. Glutamatergic System

As Tsc1–Tsc2-mediated changes in mTOR activity affect both axon formation and guidance, i.e., the presynaptic site, as well as spine shape and stabilization, i.e., the postsynaptic site, one can expect that Tsc1–Tsc2 deletion can influence synaptic transmission.

Whole cell recordings in hippocampal slice cultures with sparse conditional deletion of Tsc1 revealed that after 10 days in vitro (DIV) the miniature excitatory postsynaptic currents (mEPSC) amplitude was increased by 20%, without changes in mEPSC frequency. A potentiation of AMPAR-mediated current at the postsynaptic site was observed together with increased AMPA/NMDA ratio, while presynaptic release probability was not affected. Interestingly, at 10 DIV, membrane resistance was comparable between the two groups, but at 20 DIV it was significantly reduced in Tsc1 deficient neurons as compared to WT ones, which could represent a reduction of excitability to compensate the sustained elevation of glutamatergic inputs [70]. However, in acute brain slices of the somatosensory cortex of Tsc1^+/−^ mice around P15 no difference in the amplitude of AMPA-mediated spontaneous excitatory postsynaptic currents (sEPSCs) was detected, while the duration of sEPSC was significantly prolonged in heterozygous neurons. This change resulted from over-expression of GluN2C/D subunit of NMDA receptors. As GluN2C-mediated currents are relatively slow, this change leads to excessive temporal summation and spontaneous seizures at P9–P18. No differences in AMPA-mediated mEPSC amplitude or frequency was detected in the Syn1-Cre;Tsc1^fl/fl^ mouse at P28–30 in the visual cortex, despite a reported reduction in the amount of GluA1 and GluA2 subunits in the whole brain [74]. A similar reduction of GluA1 and GluA2 expressions led to the reduction of both mEPSC amplitude and frequency in Tsc1^−/−^ cultures [75]. Pyramidal neurons in the medial prefrontal cortex (mPFC) of Tsc2^+/−^ mice demonstrated no difference in the amplitude of AMPA-mediated mEPSCs as compared with controls, while mEPSC frequency was increased at P15–P40, suggesting a potentiation of glutamatergic drive in this mouse model [76,77]. In contrast to the mPFC, a decrease in both mEPSC amplitude and mEPSC frequency has been reported in the somatosensory cortex of Tsc2^+/−^ mice around P15. At the same time, eEPSC amplitudes were not significantly different, indicating constant strength of the glutamatergic drive [78]. In the hippocampus, no changes in evoked field potential amplitude at Schaffer collaterals-CA1 synapses was observed in both Tsc2^+/−^ rats [79] and mice [41,80]. Taken together, the finding suggests that glutamatergic synaptic transmission is altered in mice with reduced Tsc1–Tsc2 activity, but the effect might be area- as well as time-specific.

Using a novel proteomic approach reduced expression of NMDAR2B and elevated level of mGluR5 have been reported in Tsc2^+/−^ mouse model ([81], see also [82]). Activation of both NMDA and mGluR receptors can contribute to induction of long-term potentiation (LTP) and/or long-term depression (LPD), and indeed changes in synaptic plasticity were reported in Tsc1–Tsc2 deficient mice and rats. Levels of both LTP and LTD induced in acute slices by different stimulation patterns were significantly reduced in Tsc2-deficient rats [79]. Interestingly, the reduced LTP observed in Tsc2^+/−^ mice could be rescued by selective blockade of extrasynaptic NMDA receptors. This treatment could also significantly improve behavioral deficits, indicating NMDA-receptor-mediated contribution to the above changes [83]. Similarly to the reported LTP reduction, mGluR-mediated LTD was reduced in Tsc2^+/−^ mice [80]. Interestingly, using the same mouse model, Ehninger and colleagues reported no change in paired-pulse plasticity and early-LTP strength, but the level of late-LTP induced via tetanic stimulation was higher in Tsc2^+/−^ mice as compared to controls [41]. Similarly, treatment of Tsc2^+/−^ mice with mGluR5 modulators, both agonists and antagonists, has been shown to affect brain excitability. mGluR5 inhibition reduced hyperexcitability, while mGluR5 stimulation resulted in epileptic seizures [84]. Interestingly, the reduced mGluR-dependent LTD in younger (P21–25) Tsc2^+/−^ mice, showed a developmental compensation, and is not detectable at 2 months of age [85]. As for the changes in glutamatergic synaptic transmission, also plasticity shows a complex picture of developmental and region-specific regulation.

## 6. GABAergic System

Accumulating evidence suggests that mental disorders, like ASD, may be caused by changes in GABAergic transmission [86]. Reduction of both GAD65/67 and GABA_A_ receptor subunit expression has been reported in postmortem tissue samples of ASD patients [87,88]. Similarly, alpha1-GABA_A_ receptor expression was reduced in tuberal tissue resected from TSC patients, even if the frequency of sIPSCs was increased [89]. Down-regulation of alpha1 subunit of GABA_A_ receptors was shown to precede epilepsy manifestation and alpha 1 overexpression in mice reduced the incidence of spontaneous seizures by 60% [90,91]. Patch-clamp recordings of spontaneous synaptic activity performed ex vivo from human samples obtained from resection in cases of pharmacoresistant epilepsy, including TSC cases, demonstrated an increase in GABAergic spontaneous synaptic activity, but no changes in glutamatergic transmission [92].

The inhibitory action of GABA via ionotropic GABA_A_ receptors is determined not only by the number of activated receptors, but also by the transmembrane Cl^−^ gradient set by the activity of NKCC1 and KCC2 transporters [93]. As KCC2 is expressed later during development as compared to NKCC1, immature neurons have higher intracellular Cl^−^ concentration and GABA action can be depolarizing [94]. In tuberal tissue resected from TSC patients the ratio NKCC1 to KCC2 was significantly increased, indicating a potential shift of the reversal potential of GABA_A_R-mediated currents to more positive values. Perforated patch-clamp recordings confirmed that GABA_A_R-mediated currents were indeed depolarizing, indicating an impaired inhibition [89]. However, perforated patch recordings performed in the mPFC of Tsc2^+/−^ mice did not show any change of intracellular Cl^−^ concentration from P10 to P30. In addition, mIPSC properties did not show any dependence on development or genotype in WT and Tsc2^+/−^ mice. In Tsc2^+/−^ mice, phasic GABAergic transmission, both spontaneous and miniature (s/m) IPCS frequency, was strongly increased, while the strength of tonic GABA_B_R-mediated inhibition was significantly reduced, indicating a shifted balance between phasic and tonic GABAergic inhibition in Tsc2^+/−^ mice [76].

A reduction of surface alpha1-GABA_A_ receptor subunit has been reported in the visual cortex of Tsc1^c/c^; SynI-CKO mice at P28-P30. This change did not affect either mIPSC amplitude or kinetics, but caused a decrease in the frequency of mIPSCs in layer 2/3 pyramidal neurons. Surprisingly, selective deletion of Tsc1 in either parvalbumin (PV) or somatostatin (SST) interneurons failed to reproduce the observed reduction of mIPSC frequency, suggesting the involvement of other interneurons [74] or reflecting GABAergic cell reprogramming. Embryonic Tsc1 deletion in medial ganglionic eminence (MGE)-derived SST interneurons led to expression of PV and development of fast-spiking properties typical for PV interneurons. Interestingly membrane resistance was decreased in Tsc1-KO SST neurons, but their firing pattern was accelerated. Selective expression of voltage-gated K^+^ channels (Kv3.1) underlies the observed changes in firing properties [95]. Deletion of only one copy of Tsc1 in MGE-derived inhibitory cells in the hippocampus increased mTOR activity, but failed to affect either amplitudes or frequencies of both miniature glutamatergic and GABAergic currents. Although spontaneous neuronal activity was unchanged, optogenetically-induced inhibitory currents in pyramidal cells were significantly reduced in Tsc1^+/−^ mice, indicating reduced inhibition of principal cells. This change strongly affected both contextual fear discrimination and spatial working memory [44]. Thus, similar to glutamatergic system inhibitory synaptic transmission demonstrate both temporal and region dependent changes in mice with reduced Tsc1–Tsc2 activity.

## 7. Excitation/Inhibition Balance

Given the heterogeneity of genetic factors that can cause ASD, it was hypothesized that a common functional variation of neuronal activity in the CNS during development, namely a disproportion between excitatory and inhibitory drive, can contribute to ASD manifestation. Based on this hypothesis an increase in excitation could have similar functional consequences as a decrease in inhibition, i.e., leading to increased noise in key circuits responsible for computation [96]. Indeed, a similar pattern has been observed in different models of TSC, for example, decreased frequency of mIPSCs without a paired increase of mEPSC frequency both in layer 2/3 pyramidal neurons in the visual cortex of Tsc1^+/−^ mice at P30 [74] and in hippocampal cultures [97], indicating a shift of E/I balance towards excitation. Similar results have been reported in mice with genetic inactivation of Tsc1 in a subset of hippocampal CA1 neurons in vivo, which featured strongly elevated mEPSC frequency [98]. Although originally the increased excitability had been proposed to originate from the potentiation of the excitatory system, follow up investigation suggested instead another source. In fact, the Tsc-deficient cells also displayed reduced intrinsic excitability which could effectively shunt the effects of increased excitatory synaptic transmission on the network activity. The same group reported that GABAergic transmission is reduced in the CA1 pyramidal neurons after deletion of Tsc1. Based on this, the authors proposed that the increased glutamatergic synaptic transmission could have actually been a secondary effect, and the observed increase in the E/I ratio reflected rather a reduction in inhibitory transmission, showing how increased activity in mTOR pathway can lead to a cascade of induced changes and consequent adjustments, which renders the investigation complicated [75].

The existence of multiple critical time-windows has been directly shown while investigating the effects of rapamycin on dendrite arborization and spine number. Tsc1 expression in a small fraction of progenitor cells was suppressed via in utero electroporation. By the end of the first postnatal month Tsc1-suppressed cells showed more complex dendrite branching and higher spine density. Interestingly, dendrite arborization was normalized by rapamycin treatment between P1 and P13 but not after P15, while abnormal spine stabilization was achieved by rapamycin treatment between P15 and P28, but not before P13 [99]. Similarly, Tsc1^+/−^ mice showed spontaneous epileptic seizures only transiently in a specific time window (before P19). Epileptic seizures are generated intracortically. Overexpression of GluN2C of NMDA receptors appeared to underlie the observed phenomenon. In particular, the slower kinetics of synaptic currents mediated by GluN2C-containing NMDA receptors resulted in excessive temporal summation and hyperexcitability [37]. Interestingly, cortical hyperexcitability seemed to be compensated after P19 and no further epileptic seizures were detected. This time period coincides with the period of maturation and integration of parvalbumin interneurons.

Using electrophysiological recordings of postsynaptic currents functional E/I ratio has been investigated in Tsc2^+/−^ mice at P17–P23. In the somatosensory cortex a decrease in both mEPSC and mIPSC frequencies was reported, but overall the E/I ratio was shifted towards excitation [78]. In the same mouse model at P15–P19 the E/I ratio was elevated in the mPFC, but arising from selective potentiation of glutamatergic transmission [76]. In contrast to the hyperexcitability observed before P19 in Tsc1^+/−^ mice [37], sensory-evoked spiking was not increased in Tsc2^+/−^ mice. The authors hypothesize that elevated E/I ratio represents a compensatory tuning to stabilize the neuronal network [78].

Supporting this hypothesis, the increase in E/I ratio at a comparable age was not associated with an increased excitability in the mPFC. At a later time point (P25–P30) a strong potentiation in GABAergic synaptic transmission was observed (also in WT mice). Interestingly, in Tsc2^+/−^, the magnitude of this increase was scaled to balance the sustained increase in glutamatergic transmission, therefore bringing the E/I ratio to WT levels. However, at this time point, in contrast to the reduced membrane resistance in hippocampal Tsc1 KO neurons observed at about P30 [75], layer 2/3 pyramidal neurons of the mPFC displayed increased membrane resistance, and decreased tonic GABA_B_R-mediated inhibition. This might represent a maladaptive compensatory mechanism, as it unbalances the network.

Furthermore, after P30, both postsynaptic and presynaptic GABA_B_Rs were influenced in the Tsc2^+/−^ mice and ambient GABA-mediated control of GABAergic synapses but not glutamatergic ones differed in WT and Tsc2^+/−^ mice [76,77]. As extracellular GABA concentration is regulated locally by GABA transporters, astrocytes could play a pivotal role in the tonic modulation of glutamatergic and GABAergic synaptic transmission. In fact, WT neurons cultured in astrocyte-conditioned medium of astrocytes derived from TSC-patient stem cells showed an increased number of GABAergic synapses [65]. The strength and the target of glial influence on neuronal functions appears to depend on the timing of changes. For instance, deletion of either Tsc1 or Tsc2 in differentiated astrocytes did not produce tuber-like lesions (and nevertheless resulted in seizures), but inactivation of Tsc1 or Tsc2 in undifferentiated radial glia cells early during development resulted in phenotypic neuropathological features characteristic for TSC patients, including aberrations in cortical cytoarchitecture as well as epileptic seizures ([100], for review [101]).

To summarize, both Tsc1 and Tsc2 heterozygous mice appear to have a critical window during early postnatal development, which features increased excitability and imbalance between excitation and inhibition and might affect development. The early occurrence and potentially transient nature of such phenomenon raises the question of whether a short-lasting change in excitability during early postnatal development could be related to behavior deficits observed in adult animals. Recent work supports this view, e.g., transient optogenetic activation of layer 2/3 pyramidal neurons in the mPFC in vivo had been applied for five consecutive days (between P7 and P11). The immediate effect was an elevation of firing rates in about 20% of neurons, which led to working memory and social deficits in adult mice, showing directly that transient E/I disbalance indeed caused long term effects that became manifest much later [102]. 

## 8. Therapeutical Perspectives

TSC causes functional alterations which are distributed across multiple organs. Neurological manifestations such as seizures are a cause of mortality and a risk factor for further complications [29,103]. Moreover, because epilepsy prevalence in TSC patients is more than 80% and early life seizures do have a strong effect on development and intellectual disability in TSC individuals (see [104]), preventive abolishment of epileptic seizures is a crucial point in TSC treatment [105].

One possibility to reduce epileptic activity is to increase inhibition, and potentially also contrasting the increased E/I ratio. Vigabatrin, a GABA transaminase antagonist, has been administered as a disease-modifying treatment [106]. As GABA transaminase is responsible for the degradation of GABA, its vigabatrin-mediated blockade increases GABA levels and potentiates inhibition, before the onset of epilepsy. Recent study demonstrated that such preventive vigabatrin treatment in infants with TSC ameliorated several aspects, including intellectual functioning [107]. The protective effect on cognitive abilities might originate from the reduction in seizures albeit the special efficacy of vigabatrin in TSC could partially be also due to its ability to directly inhibit the mTOR pathway [108]. On the other hand, vigabatrin treatment seems to not have a strong effect on ASD symptoms [30,109].

Given the impaired chloride homeostasis in TSC patients, bumetanide, a NKCC1 blocker, was investigated, since it showed promising results in ASD treatment [110,111]. Bumetanide-induced effects were somehow opposite to that of vigabatrine, i.e., useful for treatment of ASD symptoms, but limited for seizure control [112,113].

In addition to various antiepileptic drugs, current standard treatments for epilepsy in case of TSC include direct inhibition of mTOR pathway activity with everolimus (for review [114]). The effect of rapamycin in mouse models, can be very acute, as administration for two consecutive days affect social behavior in Tsc1^+/−^ and Tsc2^+/−^ mice [39]. In Tsc1 GFAP CKO mice, mTOR pathway inhibition with rapamycin starting from 6 weeks of age, i.e., subsequent to the onset of seizures, reduced seizures within a week, greatly improving survival, as compared to the matched untreated group. However, after three weeks of treatment, astrogliosis and increased brain size compared to control mice have been observed [115]. This advocates for a mechanism which has a faster time scale than morphological changes, for example modulation of the network activity level. Similarly, everolimus as an adjuntive treatment in humans produces relatively fast effects. For example, at dosages of 9–15 ng/mL led to a reduction of circa 20% in median change in seizure frequency as compared to baseline levels after 2 weeks, with further improvements over time (up to ca 50%) [116]. Interestingly, everolimus appeared to maintain efficiency in seizure control in adult individuals, showing little age dependence in its effect, contrary to the expectation that it would provide diminished effects in later developmental stages [117]. Its use at very early ages has not been completely investigated yet, but everolimus treatment has been proven safe to use in TSC individuals under the age of 2 years, providing preliminary indication on its efficacy, despite the relatively small sample size [118]. However, in a recent study, 12 month-long everolimus treatment did not show effects on IQ, ASD symptoms and various neuropsychological features (including social functioning, executive functioning, quality of life, and sensory processing) in a randomized double-blind study when used between 4 and 17 years [119]. Early surgical intervention has also been shown to have an effect on intellectual functioning and quality of life of patients and can have a protective effect on ASD symptoms and with neurodevelopment [120].

In conclusion, the reviewed literature suggests that early intervention is crucially important in controlling seizures in TSC patients. Further, it highlights a potential segregation in the mechanism leading to seizures and ASD symptoms, as indicated by the difference in effects of vigabatrin and bumetanide. While timing appears to be an important factor to control the symptoms, such as the neurological sequelae of early epilepsy, some effects do not appear to depend strongly on age, e.g., the effect of mTOR inhibition on seizures.

## 9. Conclusions

**Figure 3:**
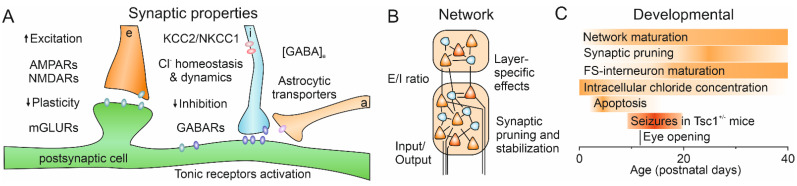
Main features that can be affected by decreased function of TSC1 or TSC2. (**A**) Synaptic properties of both excitatory and inhibitory transmission are influenced. Astrocytic control of synaptic transmission depends on mTOR pathway activity, thus potentially altering the extracellular concentration of neurotransmitter, e.g., GABA. Synaptic plasticity is altered in animals lacking TSC1 or TSC2. Not all of those alterations are necessarily observed simultaneously in terms of both brain regions and developmental stage. See text for details. e—excitatory synaptic terminal, i—inhibitory synaptic terminal, a—astrocytic process. Arrows indicate corresponding variations. (**B**) E/I ratio changes during early development may have a crucial importance for the formation of neuronal networks, including synaptic inputs and outputs. Particular interest was placed in the investigation of E/I ratio, as well as input and output. Furthermore, reduced synaptic pruning and/or strengthened spine stabilization results in an increased number of synaptic contacts. Further investigation should highlight how those changes happen in different layers and how communication within the column and across regions is affected. (**C**) During early postnatal development several processes overlap, including the maturation of the network, pruning, the maturation of fast spiking interneurons, a decrease in intracellular chloride concentration, and the second wave of apoptosis. Alterations during this time period, such as the transient seizures observed in Tsc1^+/−^ mice, might have long-term consequences. Furthermore, different brain regions undergo developmental changes at different time points, thus suggesting the possibility that alterations in synaptic or network properties might not be synchronous across the brain, and might potentially have different functional relevance.

To conclude, tuberous sclerosis is a multilevel disorder, which arises from the interplay of several factors, starting from a genetic mutation, which leads to deregulated growth and in turn gives rise to aberrant circuit structure and/or activity (Figure 3). These conditions foster the development of epilepsy, as well as ASD and TANDs. The causal links between different steps could provide a valuable topic for future investigations and also help identify potential targets for therapeutical treatments. Murine models of monogenic syndromes with high association with ASD represent a valuable tool for expanding our understanding of different aspects of this disease. However, such investigations should be conducted with the precaution of comparing results obtained in different and heterogeneous mouse models. It is of particular importance to uncover shared features that occur during early development, and to comprehend mechanisms underlying the later manifestation of symptoms. Our current knowledge indicates that occurrence of some symptoms may be region-specific. We hope that future longitudinal investigations will shed light on the functional changes in different CNS regions and will help to untangle important critical periods for effective medication.

## Figures and Tables

**Table 1:** Genetic models with decreased function of Tsc1 or Tsc2 in mice. Constitutive and conditional models lacking Tsc1 or Tsc2 genes are listed together with a brief summary of findings concerning morphology, behavior, synaptic transmission, and lethality. For readability, we specify which gene is conditionally deleted (indicated by ^c^) and the specific promoter. For a more detailed description, please refer to the related publications. Tsc1^+/−^ and Tsc2^+/−^ models have been more frequently used, see text for references. P stands for postnatal day.

Model/Specificity	Morphology and Behavior	Synaptic Transmission	Lethality
Tsc1^+/−^	No major defects Cognitive/behavioral deficits	Transient spontaneous seizures (P9–P18) Altered synaptic transmission	
Tsc1^c/−^; SynI-CKO Neurons [48]	Morphological alterations Myelination deficits	Seizures	Early death (median survival 35 days)
Tsc1^c/c^; Dlx5/6-CKO Interneurons [49]	Morphological alterations Cellular clusters in superficial layers	Reduced seizure threshold	Early death (40% by P30, 60% by P130)
Tsc1^c/+^; Nkx2.1-CKO MGE-derived interneurons [44]	Cognitive deficits	Decreased inhibition on hippocampal pyramidal cells	
Tsc1^c/c^; Nestin-CKO (rtTA^+^ TetOp-Cre^+)^ Neural progenitors [50]	Increased brain size Giant cells in hippocampus and cortex	Spontaneous seizures (around 3rd week)	Early death
Tsc1^c/c^; Emx1-CKO Dorsal neural progenitors [51]	Increased brain size and cortical thickness Lamination deficits Subependymal nodules Astrocytosis Myelination deficits	Spontaneous seizures (100% of mutants at P13)	Early death (median survival 18 days)
Tsc1^c/+^ and Tsc1^c/c^; L7 CKO Purkinje cells (PC) [52]	Decreased PC number Behavioral deficits	Increased spine density and decreased excitability in PC	
Tsc1^c/c^; Gbx2 CKO (CreER) Thalamus Tamoxifen at E12 or E18 [53]	Morphological alterations (stronger for earlier deletions), Behavioral deficits	Spontaneous seizures Altered active properties of thalamic neurons for early deletions	
Tsc2^+/−^	No major morphological defects Cognitive/behavioral deficits	Altered synaptic transmission and plasticity	
Tsc2^c/−^; HGFAP CKO Radial glia progenitors [54]	Increased brain size and cortical thickness Lamination deficits	Possible seizures	Early death (by 4^th^ week)
Tsc2^c/c^; Emx1 CKO Dorsal neural progenitors [55]		Spontaneous seizures	Early death (by 3^rd^ week)
Tsc2^c/c^; Nex CKO Postmitotic excitatory forebrain neurons [56]	Alterations in neurons and glia Minor lamination defects in hippocampus and deep layers Astrogliosis		Early death (0% survival P22)
Tsc2^c/c^; Olig2 CKO Oligodendrocyte precursor cells [57]	Hypomyelination Astrogliosis	No seizures	
Tsc1 GFAP1 CKO and Tsc2 GFAP1 CKO GFAP-positive cells [58]	Increased brain size Astrocytic proliferation Structural alterations of the hippocampus	Spontaneous seizures (onset: 4 wks for Tsc1 and 3 wks for Tsc2) Decreased glutamate transporter expression	Early death (Tsc1: 50% survival at 9 wks, 0% at 18 wks; Tsc2: 50% survival at 7 wks, 0% at 10 wks)
Tsc1^fl/−^, Tsc2^fl/−^ and Tsc1^fl/−^;Tsc2^fl/−^; FVB-Tg(GFAP-cre)25Mes/J CKO Radial glia [59]	Macrocephaly Lamination deficits Astrogliosis Myelination and oligodendrocyte alterations	Possible seizures	Early death (50% death at around P20–23)
